# Phase 1 dose escalation study of the MDM2 inhibitor milademetan as monotherapy and in combination with azacitidine in patients with myeloid malignancies

**DOI:** 10.1002/cam4.70028

**Published:** 2024-07-19

**Authors:** Courtney D. DiNardo, Rebecca Olin, Eunice S. Wang, Barry Skikne, Joseph Rosenthal, Prasanna Kumar, Hiroyuki Sumi, Yoshiyuki Hizukuri, Ying Hong, Parul Patel, Takahiko Seki, Tao Duan, Arnaud Lesegretain, Michael Andreeff

**Affiliations:** ^1^ Department of Leukemia The University of Texas MD Anderson Cancer Center Houston Texas USA; ^2^ University of California San Francisco California USA; ^3^ Roswell Park Comprehensive Care Center Buffalo New York USA; ^4^ University of Kansas Medical Center Kansas City Kansas USA; ^5^ Department of Pediatrics City of Hope Duarte California USA; ^6^ Daiichi Sankyo Inc. Basking Ridge New Jersey USA; ^7^ Daiichi Sankyo Co. Tokyo Japan

**Keywords:** acute myeloid leukemia, milademetan, mouse double minute‐2 homolog, myelodysplastic syndromes

## Abstract

**Background:**

Mouse double minute‐2 homolog (MDM2) plays a key role in downregulating p53 activity in hematologic malignancies, and its overexpression is associated with poor outcomes.

**Methods:**

This phase 1 study assessed the safety and efficacy of different dosing regimens of the MDM2 inhibitor milademetan as monotherapy and in combination with azacitidine (AZA) in patients with relapsed or refractory acute myeloid leukemia or high‐risk myelodysplastic syndromes.

**Results:**

Seventy‐four patients (monotherapy, *n* = 57; milademetan‐AZA combination, *n* = 17) were treated. The maximum tolerated dose of milademetan was 160 mg once daily given for the first 14–21 days of 28‐day cycles as monotherapy and on Days 5–14 in combination with AZA. Dose‐limiting toxicities were gastrointestinal, fatigue, or renal/electrolyte abnormalities. Treatment‐emergent adverse events related to milademetan occurred in 82.5% and 64.7% of participants in the monotherapy and AZA combination arms, respectively. Two participants (4.2%) in the monotherapy arm achieved complete remission (CR), and 1 (2.1%) achieved CR with incomplete blood count recovery (CRi). Two participants (13.3%) achieved CRi in the combination arm. New *TP53* mutations, detected only during milademetan monotherapy, were found pre‐existing below standard detection frequency by droplet digital polymerase chain reaction.

**Interpretation:**

Milademetan was relatively well tolerated in this population; however, despite signals of activity, clinical efficacy was minimal.

## 1 INTRODUCTION

The tumor suppressor p53 is dysfunctional in most malignancies, mainly due to *TP53* mutations and negative regulatory mechanisms.^1^ *TP53* missense mutations result in altered p53 function^2^ and occur in ~10% of patients with de novo acute myeloid leukemia (AML)/myelodysplastic syndromes (MDS).^3^, ^4^, ^5^ *TP53* mutations occur even more frequently in patients with treatment‐related AML/MDS (~30%) and complex karyotype AML (60%–80%).^6^, ^7^, ^8^ *TP53* mutations are associated with significantly poorer outcomes in patients with AML/MDS, including inferior remission rates, shorter overall survival, and higher relapse rates relative to patients with wild‐type *TP53*.^3^, ^4^, ^5^, ^6^, ^7^

In the absence of *TP53* loss‐of‐function mutations, p53 signaling may be negatively regulated by various key proteins,^9^, ^10^ including binding of the mouse double minute‐2 homolog (MDM2) protein.^1^, ^11^ The binding of MDM2 leads to ubiquitination and proteasomal degradation of p53, resulting in reduced cellular levels and downregulation of p53 target genes. Notably, p53 also activates MDM2 expression, thus creating an autoregulatory feedback loop.

In *TP53* wild‐type AML, MDM2 may be overexpressed, thereby impacting p53 function.^9^, ^12^, ^13^ MDM2 overexpression in patients with AML is associated with reduced event‐free survival and a shorter remission duration.^14^ Targeting MDM2 may, therefore, reactivate p53 and provide an option for treating AML/MDS with wild‐type *TP53*.^9^, ^15^, ^16^, ^17^ A phase 1 clinical study with the MDM2 inhibitor, Nutlin‐3a analog RG7112, conducted in AML showed a response rate of ~25%.^18^

DS‐3032b (milademetan tosylate hydrate; hereafter called milademetan) inhibits the interaction between MDM2 and p53 and has shown tumor growth inhibition and induction of apoptosis in both in vitro and in vivo cancer models with wild‐type p53.^19^ Additionally, intermittent dosing of milademetan has shown clinical activity in patients with dedifferentiated liposarcomas.^20^ Here, we report the safety and preliminary efficacy results from a phase 1 dose escalation study evaluating milademetan as monotherapy and in combination with 5‐azacytidine (AZA) in patients with AML and high‐risk MDS.

## 2 METHODS

### 2.1 Study participants

Patient enrollment began on November 25, 2014, and concluded on August 21, 2020. Individuals aged ≥18 years were eligible to participate if they had relapsed or refractory AML (according to the 2016 World Health Organization criteria classification^21^) or high‐risk MDS (defined by Revised International Prognostic Scoring System score of High or Very High^22^), with or without prior treatment. Participants were required to have an Eastern Cooperative Oncology Group performance status of 0 to 2, adequate renal function (creatinine clearance ≥60 mL/min, or creatinine clearance 50–60 mL/min and serum creatinine ≤1.5 × upper limit of normal [ULN]), and adequate hepatic function (aspartate aminotransferase/alanine aminotransferase ≤2.5 × ULN [≤5 × ULN if elevated due to leukemia], and serum total bilirubin ≤1.5 × ULN [3 × ULN if elevated due to leukemia or in participants with Gilbert syndrome]).

Individuals were excluded if they had acute promyelocytic leukemia, a known *TP53*‐mutated malignancy, central nervous system involvement, a second concurrent primary malignancy treated with systemic therapy within the past 2 years, hematopoietic stem cell transplant within the previous 60 days, acute or chronic infections (including human immunodeficiency virus, hepatitis B, or C), or significant cardiovascular disease or impaired lung function. Significant cardiovascular disease involves prolongation of corrected QT interval or family history of long QT syndrome, bradycardia <50 bpm, ventricular arrhythmias, history of second‐ or third‐degree heart block, myocardial infarction or uncontrolled angina pectoris within 6 months before screening, New York Heart Association (NYHA) Class III or IV congestive heart failure, known left ventricular ejection fraction ≤50%, uncontrolled hypertension, or left bundle block. Impaired lung function was defined as patients having a known diffusing capacity for carbon monoxide (DLCO) ≤65% or forced expiratory volume in the first second (FEV1) ≤65%.

The trial was conducted in accordance with the principles of Good Clinical Practice, according to the International Council for Harmonization of Technical Requirements for Pharmaceuticals for Human Use Harmonized Tripartite Guideline. All participants provided written informed consent.

### 2.2 Study design

This was a phase 1, open‐label, non‐randomized dose escalation study of milademetan as monotherapy (Part 1) or in combination with AZA (Part 1A; ClinicalTrials.gov identifier: NCT02319369).

Dose escalation of milademetan to determine the maximum tolerated dose (MTD) was guided by a Bayesian logistic regression model (BLRM) following escalation with overdose control principle with a starting dose of 60 mg based on safety and tolerability data from the first‐in‐human study of milademetan in solid tumors or lymphoma.^20^ Dose escalation was also guided by the following restrictions: the dose‐level increment should be no less than 30% but no more than 100% to have distinction among dose levels, and treatment was continued until disease progression or unacceptable toxicity.

In Part 1, 9 escalating dose cohorts (Figure S1) were investigated. Milademetan was administered once daily (QD) for 21 days of a 28‐day cycle (QD 21/28) at doses of 60, 90, 120, 160, and 210 mg in Cohorts 1 through 5, respectively; at 160 mg QD for 7 days of a 28‐day cycle (QD 7/28) in Cohort 6; at 160 mg QD for 3 days every 14 days, given twice in a 28‐day cycle (QD 3/14 × 2) in Cohort 7; and at 160 or 220 mg QD for 14 days of a 28‐day cycle (QD 14/28) in Cohorts 8 and 9, respectively.

In Part 1A, milademetan was given in combination with AZA (75 mg/m^2^ dose administered subcutaneously or intravenously on Days 1–7) with 2 dosing schedules of milademetan: in Cohorts 10 and 12, milademetan was administered QD for 10 days (Days 5–14; 160 and 200 mg, respectively); in Cohorts 11 and 13 milademetan was given QD for 7 days (Days 8–14; 160 and 200 mg, respectively). The delayed start of miladementan dosing relative to AZA in each cycle was hypothesized to allow AZA incorporation into DNA before p53 induced cell cycle arrest, which was informed by preclinical xenograft studies (Figure S2).

### 2.3 Study outcomes/endpoints

Safety was the primary endpoint of the study and included treatment‐emergent adverse events (TEAE), serious adverse events (SAE), dose‐limiting toxicities (DLT), physical examination findings, and clinical laboratory values. TEAE were categorized according to the *Medical Dictionary for Regulatory Activities* (MeDRA) v17.0 and graded according to the National Cancer Institute‐Common Terminology for Adverse Events (NCI‐CTCAE) v5.0. Participants were followed up until 30 days after completion of study treatment. A DLT was defined as any TEAE of grade ≥3 not attributable to disease or disease‐related processes occurring during Cycle 1 in each dose‐level cohort. DLT could also include grade ≥2 TEAE leading to inability to complete ≥75% of scheduled milademetan or AZA treatment.

The MTD was estimated by BLRM and escalation with overdose control as the dose with the highest posterior probability of the DLT rate in the target DLT rate interval of [16%, 33%] and with less than 25% probability for the DLT rate >33% (probability for excessive and unacceptable toxicity). The final MTD for each dosing regimen (QD 21/28 dosing and others) was decided based on considerations of the respective MTD estimated by the BLRM and on an overall assessment of safety data from subsequent cycles and pharmacokinetics (PK)/pharmacodynamics (PD) information collected at all different doses tested.

Treatment efficacy was assessed according to the 2017 European Leukemia Net recommendations for AML^23^ and the 2006 International Working Group response criteria for MDS.^24^ In participants with AML, efficacy outcomes included complete remission (CR), CR with incomplete blood count recovery (CRi), composite complete remission (CRc; CR + CRi), morphologic leukemia‐free state (MLFS), partial remission (PR), overall response rate (CRc + MLFS+PR), stable disease (SD), and treatment failure. Bone marrow biopsies/aspirates were obtained from all participants with AML on Day 1 of Cycles 2 and 3 and once every cycle until response and on Day 1 of Cycles 6, 9, and 12 in responding participants.

For PK analysis, blood samples were collected at frequent intervals during Cycle 1 Days 1 and 15 in Part 1 (monotherapy), and on Day 5 or 8 (according to dosing schedule) and Day 14 in Part 1A, with sparse sampling on other days. Derived PK parameters for milademetan included maximum plasma concentration (*C*_max_), time to reach maximum plasma concentration (*T*_max_), trough plasma concentration (*C*_trough_), area under the plasma concentration‐time curve up to 24 h (AUC_0‐24h_) and apparent clearance of drug from plasma (CL/F).

Macrophage inhibitory cytokine‐1 (MIC‐1) is a target of p53 transcriptional activation,^25^ and, therefore, change in serum MIC‐1 levels was included as a PD endpoint, measured at multiple time points.

### 2.4 TP53 mutation status

Analyses of blood and bone marrow aspirates for *TP53* mutations was an exploratory endpoint of the study.

In Part 1 of the study, *TP53* gene region was amplified by polymerase chain reaction and sequenced by next‐generation sequencing (NGS) to detect mutations. A variant allele frequency (VAF) ≥5% was detected as somatic mutations after filtering of the known germline mutations. Due to discontinuation of TP53 NGS assay, Part 1A study samples were tested using Myeloid genomic alteration assay (VariantPlex; Integrated DNA Technologies, Coralville, Iowa) with detection sensitivity of >2.7% VAF offered by Archer. Additionally, leftover samples from Part 1 were also analyzed by Archer myeloid panel assay. A more sensitive digital droplet polymerase chain reaction (ddPCR) for common *TP53* mutations of interest (p.R248W, p.R248Q, p.R273H, p.I251N, p.V274L, and p.F134fs) was used to re‐test the baseline and post‐dose samples available from patients who showed emergent mutations during the treatment by TP53 NGS or Archer myeloid panel NGS.

### 2.5 Statistical analyses

Cohorts of ≥3 DLT evaluable participants were enrolled per dose level, and the dose escalation decisions were guided by BLRM, governed by escalation with overdose control principle. For participants to be considered evaluable for dose escalation decisions, they must have received ≥75% of the prescribed doses or experienced a DLT during the DLT evaluation period (Cycle 1).

Safety, efficacy, and PD analyses were conducted on data from all participants who had received ≥1 dose of the study drug. The PK analysis set included all participants who had received ≥1 dose of milademetan or AZA and had measurable plasma concentrations of ≥1 drug. For PK/PD analysis, the scatter plot of MIC‐1 fold change and time‐matched milademetan plasma concentration was provided for overall Part 1 and Part 1A.

## 3 RESULTS

Seventy‐four participants were enrolled and treated: 57 in the milademetan monotherapy arm (Part 1) and 17 in the milademetan and AZA combination arm (Part 1A). The median age for the total study population was 69 years, 61% were male, and 85% had AML. Fifty‐seven participants (77%) had received ≥3 prior anticancer drug therapies, and 6 participants had a prior hematopoietic stem cell transplant (Table 1).

**TABLE 1:** Demographic and baseline disease characteristics (safety analysis set).

Characteristic	Milademetan monotherapy (*N* = 57)	Milademetan + AZA (*N* = 17)	Total (*N* = 74)
Median (min, max) age, years	69.0 (30, 88)	64.0 (21, 82)	69.0 (21, 88)
Male, *n* (%)	37 (64.9)	8 (47.1)	45 (60.8)
Race, *n* (%)			
White	46 (80.7)	14 (82.4)	60 (81.1)
Black or African American	3 (5.3)	2 (11.8)	5 (6.8)
Asian	4 (7.0)	0	4 (5.4)
Other	4 (7.0)	1 (5.9)	5 (6.8)
Ethnicity, *n* (%)			
Hispanic or Latino	4 (7.0)	1 (5.9)	5 (6.8)
Not Hispanic or Latino	50 (87.7)	16 (94.1)	66 (89.2)
Not reported	3 (5.3)	0	3 (4.1)
ECOG performance status, *n* (%)			
0	8 (14.0)	2 (11.8)	10 (13.5)
1	38 (66.7)	7 (41.2)	45 (60.8)
2	11 (19.3)	8 (47.1)	19 (25.7)
Diagnosis, *n* (%)			
AML^a^	48 (84.2)	15 (88.2)	63 (85.1)
Adverse	21 (36.8)	11 (64.7)	32 (43.2)
Favorable	1 (1.8)	0	1 (1.4)
Intermediate	23 (40.4)	4 (23.5)	27 (36.5)
Unknown	3 (5.3)	0	3 (4.1)
MDS (high risk)	9 (15.8)	2 (11.8)	11 (14.9)
IPSS‐R risk: High	8 (14.0)	2 (11.8)	10 (13.5)
IPSS‐R risk: Very high	1 (1.8)	0	1 (1.4)
*TP53* genotype, *n* (%)			
Wild‐type or noninactivating mutations	55 (96.5)	16 (94.1)^b^	71 (95.9)
Inactivating mutations	1 (1.8)	1 (5.9)^b^	2 (2.7)
Indeterminate/unknown^c^	1 (1.8)	0^b^	1 (1.4)
No. of prior drug therapies, *n* (%)			
0	0	2 (11.8)	2 (2.7)
1	9 (15.8)	0	9 (12.2)
2	5 (8.8)	1 (5.9)	6 (8.1)
≥3	43 (75.4)	14 (82.4)	57 (77.0)
Prior AZA, *n* (%)	28 (49.1)	7 (41.2)	35 (47.3)
Prior decitabine, *n* (%)	26 (45.6)	12 (70.6)	38 (51.4)

^a^ According to 2017 European LeukemiaNet recommendations.

^b^ Samples in the monotherapy group were analyzed by next‐generation sequencing at Covance Genomics whereas samples in the combination group were only analyzed by Archer panel sequencing.

^c^ These were identified on retrospective review after enrollment.

Abbreviations: AML, acute myelogenous leukemia; AZA, azacytidine; ECOG, Eastern Cooperative Oncology Group; IPSS‐R, International Prognostic Scoring System‐Revised; MDS, myelodysplastic syndrome.

### 3.1 Maximum tolerated dose and dose‐limiting toxicities

Dose escalation under Part 1 started with 60 mg milademetan QD 21/28 and escalated to 210 mg QD 21/28 (Cohort 5), which exceeded MTD due to intolerable toxicities. Additional participants were then enrolled at the 120 and 160 mg doses, and 160 mg was determined to be the MTD in the QD 21/28 and QD 14/28 dosing schedules. Eight participants experienced DLT at doses of 60 mg QD 21/28 (*n* = 1), 160 mg QD 21/28 (*n* = 2), 210 mg QD 21/28 (*n* = 3), and 220 mg QD 14/28 (*n* = 2). DLT were primarily related to gastrointestinal or renal/electrolyte toxicity: 3 participants experienced nausea; fatigue, cellulitis, diarrhea, hypokalemia, renal failure, and vomiting were DLT experienced by 1 participant each (Table S1).

In Part 1A, the MTD was determined to be 160 mg milademetan (QD on Days 5 to 14) plus 75 mg/m^2^ AZA (QD on Days 1 to 7) in each 28‐day cycle. Two participants who received 200 mg milademetan plus AZA in the above schedule experienced DLT (grade >3 fatigue in both participants and syncope in 1 participant; Table S1).

All participants discontinued study treatment before the data cutoff (August 21, 2020). The most common documented reasons for treatment discontinuation included persistent or progressive disease (53% and 47%, respectively), failure to achieve response (16%), and TEAE (14%). Of note, 1 participant was discovered to have a *TP53*‐mutated malignancy after completion of genetic testing several weeks after treatment began and was discontinued due to the lack of clinical benefit. Participants in the monotherapy arm received a median (range) of 1 (1–12) treatment cycle; 19 participants (33%) initiated 2 cycles and 10 (18%) initiated 3 cycles. Participants in the AZA combination arm received a median (range) of 1 (1–20) treatment cycle, with 8 participants (47%) initiating 2 cycles and 6 (35%) initiating 3 cycles.

### 3.2 Adverse events

#### 3.2.1 Milademetan monotherapy

All 57 participants in the monotherapy arm experienced TEAE, with 48 (84%) experiencing grade ≥3 TEAE and 9 (16%) experiencing a fatal TEAE (Table S2). Dose interruptions, reductions, and discontinuations due to TEAE occurred in 12 (21%), 3 (5%), and 13 (23%) participants, respectively. Forty‐seven participants (82%) experienced milademetan‐related TEAE per investigator assessment, including 19 (33%) with grade ≥3 TEAE and 7 (12%) with SAE. The most common (>20%) TEAE (Table 2) were related to the digestive tract: nausea (*n* = 32 [56%]), diarrhea (*n* = 25 [44%]), and vomiting (15 [26%]). Augmented prophylactic use of antidiarrheal and antiemetic medications were able to abrogate the frequency and intensity of these TEAE.

**TABLE 2:** Most frequently reported treatment‐emergent adverse events (≥10% of participants; safety analysis set).

TEAE, *n* (%)	Milademetan monotherapy (*N* = 57)		Milademetan + AZA (*N* = 17)	
	All grades	Grade ≥3	All grades	Grade ≥3
Any TEAE	57 (100)	48 (84)	17 (100)	16 (94)
Nausea	39 (68)	4 (7)	10 (59)	1 (6)
Diarrhea	32 (56)	3 (5)	4 (24)	1 (6)
Vomiting	24 (42)	2 (4)	5 (29)	0
Fatigue	20 (35)	4 (7)	7 (41)	3 (18)
Thrombocytopenia	15 (26)	15 (26)	3 (18)	3 (18)
Peripheral edema	14 (25)	0	3 (18)	0
Decreased appetite	13 (23)	1 (2)	5 (29)	0
Anemia	13 (23)	9 (16)	1 (6)	1 (6)
Hypokalemia	12 (21)	3 (5)	2 (12)	0
Lung infection	11 (19)	11 (19)	6 (35)	6 (35)
Neutropenia	10 (18)	10 (18)	2 (12)	2 (12)
Hypomagnesemia	9 (16)	0	1 (6)	0
Hypotension	9 (16)	2 (4)	5 (29)	1 (6)
Pneumonia	9 (16)	6 (11)	5 (29)	4 (24)
Dyspnea	8 (14)	0	6 (35)	0
Sepsis	8 (14)	8 (14)	2 (12)	2 (12)
Abdominal pain	7 (12)	1 (2)	5 (29)	1 (6)
Asthenia	7 (12)	1 (2)	0	0
Dehydration	6 (11)	1 (2)	2 (12)	0
Dizziness	6 (11)	1 (2)	4 (24)	1 (6)
Febrile neutropenia	6 (11)	5 (9)	1 (6)	1 (6)
Hyperuricemia	6 (11)	2 (4)	0	0
Malaise	6 (11)	0 (0)	0	0
Pyrexia	5 (9)	1 (2)	2 (12)	0
Insomnia	4 (7)	0	2 (12)	0
Arthralgia	3 (5)	0	2 (12)	0
Cough	3 (5)	0	7 (41)	0
Constipation	3 (5)	0	6 (35)	0
Hemorrhoids	3 (5)	1 (2)	2 (12)	0
Oropharyngeal pain	3 (5)	0	2 (12)	0
Contusion	2 (4)	0	4 (24)	0
Depression	2 (4)	0	2 (12)	0
Fluid overload	2 (4)	0	2 (12)	1 (6)
Fungal pneumonia	2 (4)	2 (4)	2 (12)	2 (12)
Headache	2 (4)	0	3 (18)	0
Hyponatremia	2 (4)	2 (4)	2 (12)	0
Muscular weakness	2 (4)	0	3 (18)	0
Maculopapular rash	2 (4)	0	3 (18)	0
Epistaxis	1 (2)	0	3 (18)	1 (6)
Increased ALT	1 (2)	0	2 (12)	1 (6)
Myalgia	1 (2)	0	4 (24)	0
Rhinorrhea	1 (2)	0	3 (18)	0
Device‐related infection	0	0	2 (12)	2 (12)
Escherichia infection	0	0	2 (12)	1 (6)
Hypophosphatemia	0	0	3 (18)	0
Pancytopenia	0	0	2 (12)	0

Abbreviations: ALT, alanine aminotransferase; AZA, azacytidine; TEAE, treatment‐emergent adverse events.

SAEs in the monotherapy arm were primarily infectious in nature—lung infection (*n* = 9 [16%]); pneumonia (*n* = 7 [12%]); sepsis (*n* = 7 [12%]); febrile neutropenia (*n* = 5 [9%]); and bacteremia, cellulitis, and pyrexia (*n* = 3 [5%] each) and reflected the longstanding immunodeficiency in many relapsed/refractory AML/MDS patients. Of the seven miladementan‐related SAE, three were treated with the 210‐mg dose (>MTD; Table S1). There were no deaths related to milademetan treatment.

#### 3.2.2 Milademetan and AZA combination therapy

All 17 participants in the combination arm experienced TEAE, with grade ≥3 TEAE and SAE reported in 16 (94%) each and fatal TEAE in 5 (29%; Table S2). Four participants (24%) discontinued milademetan and 3 (18%) discontinued AZA treatment due to TEAE. Milademetan‐related TEAE and SAE occurred in 11 (65%) and 4 (24%) participants, respectively, with grade 3 TEAE in 5 participants (29%). AZA‐related TEAE and SAE occurred in 15 (88%) and 3 (18%) participants, respectively, with grade 3 TEAE in 7 participants (41%). The most frequent milademetan‐related TEAE included nausea (*n* = 9 [53%]), fatigue (*n* = 5 [29%]), and vomiting (*n* = 3 [18%]). As in the monotherapy arm, the most common SAE in the combination arm included lung infection (*n* = 6 [35%]), pneumonia (*n* = 4 [24%]), fungal pneumonia (*n* = 2 [12%]), and sepsis (*n* = 2 [12%]). Grade ≥3 anemia, neutropenia, and thrombocytopenia occurred in 1 participant each. There were no deaths associated with milademetan or AZA treatment.

### 3.3 Efficacy

**FIGURE 1:**
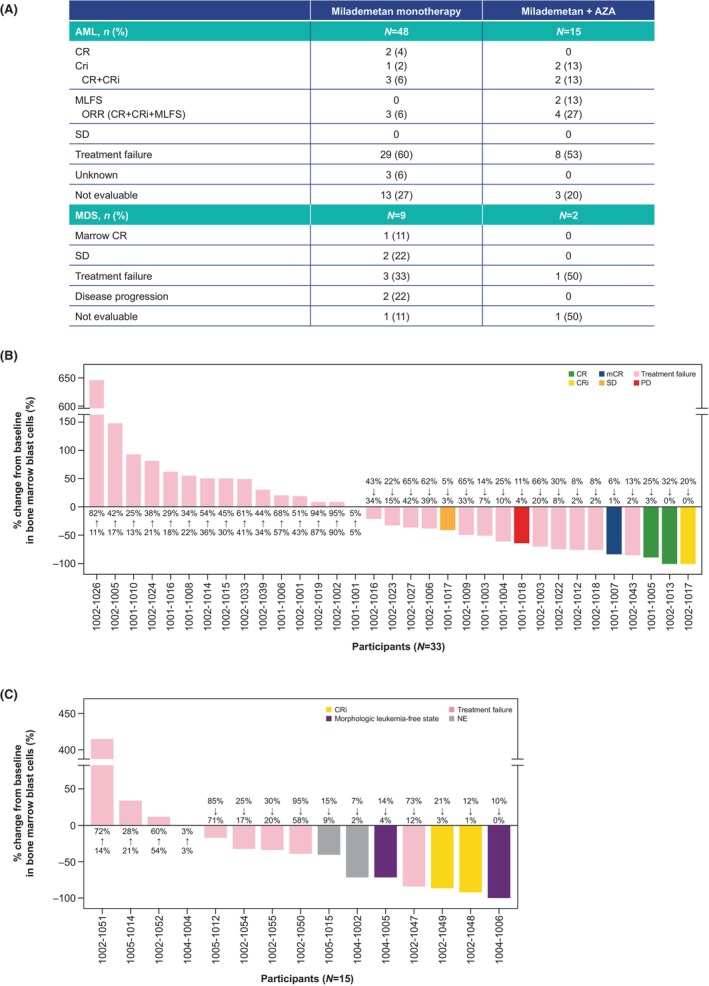
Treatment response (A) and waterfall plots for percentage change from baseline in bone marrow blast cells for (B) milademetan monotherapy and (C) combination milademetan and azacytidine (Full Analysis Set). AML, acute myeloid leukemia; AZA, azacytidine; CR, complete remission; CRi, complete remission with incomplete hematological recovery; mCR, marrow complete response; MDS, myelodysplastic syndrome; MLFS, morphologic leukemia‐free state; NE, not evaluable; ORR, overall response rate; PD, progressive disease; SD, stable disease.

Among the 48 participants with AML in Part 1 (monotherapy), 2 (4%) achieved CR, 1 (2%) achieved CRi, and the remainder had treatment failure, were not evaluable, or status was unknown. Among the participants with high‐risk MDS, 1 (11%) achieved a marrow complete response (mCR) and 2 (22%) had SD; the majority of the remaining participants had treatment failure or were not evaluable (Figure 1A).

In Part 1A (AZA combination), 2 (13%) of the 15 participants with AML had CRi, 2 (13%) had MLFS, 8 (53%) had treatment failure and 3 (20%) were not evaluable. In the 2 participants with MDS, 1 had treatment failure and 1 was not evaluable. Waterfall plots showing best responses are presented in Figure 1B,C. Baseline characteristics of participants with response or SD are presented in Table S3.

### 3.4 Milademetan pharmacokinetics and pharmacodynamics

Milademetan concentration‐time curves for all monotherapy cohorts are presented in Figure S3. In the milademetan monotherapy arm, on Cycle 1 Day 1, *C*_max_ (power model estimated slope, 0.76; 90% CI, 0.44–1.08) and AUC_0‐24h_ (power model estimated slope, 0.82; 90% CI, 0.49–1.15) increased linearly with dose (Figure S4). For all the studied doses of milademetan monotherapy, on Cycle 1 Day 1, *T*_max_ was between 3.0 and 4.6 h, the geometric mean t_1/2_ ranged between 12.9 to 24.6 h, and the estimated CL/F was 6.45 to 15.48 L/h.

On Cycle 1 Day 15, the geometric mean of the t_1/2_ was between 7.2 and 25.2 h, and steady state CL/F was between 8.28 and 20.74 L/h, suggesting that milademetan reaches steady state by Day 8 following repeated daily doses. After the multiple doses of milademetan 160 mg QD for 15 days in Cycle 1, the accumulation ratio (AR), calculated based on *C*_max_, was 1.21 and AR based on AUC_0‐24h_ was 1.29.

For all cohorts in the monotherapy arm, mean (standard deviation; SD) serum MIC‐1 at baseline was 4044.4 pg/mL (4074.3). The largest mean (SD) fold change from baseline during Cycle 1 was observed on Day 8 (*N* = 53) at 6.4 (5.8) and Day 15 (*N* = 49) at 5.1 (4.2). By Cycle 1 Days 21 through 22, the mean (SD) fold change was 4.1 (3.3). There was an increase in serum MIC‐1 concentration with increasing plasma milademetan concentrations at all‐time points (Figure S5).

### 3.5 TP53 mutation status

In 4 of the 57 participants (7%) receiving milademetan monotherapy, 2 of whom achieved CR and 2 who had treatment failure, *TP53* mutations were detected by TP53 PCR/NGS or Archer myeloid panel NGS on Cycle 2 Day 1 or end of treatment, with no mutations detectable at baseline (Table S4 and Table 3). In one additional participant who had a detectable *TP53* mutation at baseline, the VAF of the same mutation increased from 69% at baseline to 99% by NGS on Cycle 2 Day 1. Among the two participants who achieved a CR with milademetan monotherapy and had emergent *TP53* mutations, one (Id: 10011005) had emergent R248Q [VAF 16%], as well as R248W [VAF 28%] at end of treatment by TP53 NGS, and the other (Id: 10021013) had emergent R273H [VAF 4%] at Cycle 2 Day 1 by Archer Myeloid Panel NGS. When samples available from two of these four participants with emergent *TP53* mutations were analyzed by a more sensitive ddPCR, the same mutations were indeed present at low pretreatment levels, suggesting that pre‐existing clones expanded despite milademetan monotherapy (Table 3; Id: 10011005 and 10011016). One more participant who achieved a CRi with milademetan monotherapy (Id: 10021017) had a *TP53* mutation detected at baseline (Archer Myeloid panel: 5_prime_UTR_variant not affecting p53 protein function [VAF 2.9]) but not at end of treatment. All other participants who achieved a response or SD had no *TP53* mutations either at baseline or during treatment.

**TABLE 3:** *TP53* mutations at baseline and post‐treatment.

Treatment	Patient id	Archer myeloid panel		TP53 NGS assay		ddPCR assay	
		Baseline (VAF%)	Post‐treatment (VAF%)	Baseline (VAF%)	Post‐treatment (VAF%)	Baseline (VAF%)	Post‐treatment (VAF%)
Milademetan monotherapy	10021027	5_prime_UTR_variant(79.3)	NA	NA	NA	NA	NA
	10021017	5_prime_UTR_variant(2.9)	Wild‐type	NA	NA	NA	NA
	10021024	Wild type	p.V122DfsTer26 (−10.4)	Wild‐type	p.V122fs (11)^a^	NA	NA
	10021018	Wild type	p.I251AfsTer14 (−12.7)	Wild‐type	p.P250fs (13)	NA	NA
	10011005	Wild type	p.R248Q(15.5)	Wild‐type	p.R248Q (16)	R248W (0.62%)	R248W (C2D1: 27.6%)
			p.R248W(29.3)		p.R248W (28)	R248Q (0.46%)	R248Q (C2D1: 18.2%)
						R273H (0.08%)	R273H (C2D1: 2.2%)
	10021013	Wild type	p.R273H (3.9)	NA	NA	NA	NA
	10011016	p.R213Ter(68.7)	p.R213Ter (−99.1)	p.R213*(91)	p.R213* (100)	R273H (0.18%)^b^	R273H (C1D1: 0.12%)
							R273H (C2D1: 2.6%)
							I251N (C2D1: 0.33%)
							R248Q (C2D1:0.19%)
Milademetan + AZA	10041004	p.R282W(26.6)	p.K132R (2.9)	NA	NA	NA	NA
		p.K132R(25.0)					

^a^ Less than lower limit of quantitation (12.5%).

^b^ Detected at the screening visit.

Abbreviations: AZA, azacytidine; C1D1, Cycle 1 Day 1; C2D1, Cycle 2 Day 1; ddPCR, digital droplet polymerase chain reaction; EOT, end of treatment; NGS, next‐generation sequencing; PCR, polymerase chain reaction; VAF, variant allele frequency.

In contrast with milademetan monotherapy, none of the 17 participants in the combination arm had evidence for selection/enrichment of p53 mutants as newly emergent clones. Additionally, one participant (Id: 10041004) with a loss‐of‐function *TP53* mutant (K132R) clone at baseline showed a substantial reduction in VAF (from 25% to 3%) on Cycle 2 Day 1; in this participant, another coexisting mutant (R282W: VAF 27%) clone became undetectable after treatment (Table 3).

## 4 DISCUSSION

This study evaluated the safety, efficacy, PK, and PD of milademetan as a single agent and in combination with AZA in participants with relapsed/resistant AML or high‐risk MDS. This was a heavily pre‐treated population, with 77% having received ≥3 prior anticancer regimens. Such treatment‐exposed patients have few therapy options, and novel agents are needed.

Milademetan was generally well tolerated, with a MTD of 160 mg, regardless of dosing schedule. The most common TEAE were gastrointestinal toxicities and fatigue, similar to observations in two phase 1 studies of milademetan conducted in the United States and Japan in patients with solid tumors or lymphomas^26^; in both studies, DLT of thrombocytopenia and nausea were observed at the 120 mg QD 21/28 dose. The US study in solid tumors explored more intermittent dosing schedules that showed better tolerability at higher dose levels with an MTD of 260 mg in the QD 3/14 × 2 schedule and increased efficacy.^20^ However, this intermittent dosing schedule was not effective in controlling a highly proliferative disease such as AML. Another MDM2 inhibitor in clinical development, idasanutlin, has demonstrated a comparable toxicity profile.^27^

Few responses to treatment were observed in either arm of this heavily pre‐treated population, with remission in 4 participants (7%) receiving milademetan monotherapy and 2 (13%) receiving AZA combination. A similar lack of efficacy with idasanutlin in combination with cytarabine has been shown in a large clinical trial of patients with relapsed/refractory AML and wild‐type p53^27^; an overall response rate of 39% was reported in the combination arm, compared with 22% in the cytarabine monotherapy arm, and without significant difference in terms of overall survival. Preclinical studies indicated activity of simultaneously inhibiting MDM2 and Bcl‐2 pathways in overcoming resistance to apoptosis,^28^ and the predicted efficacy was at least partially confirmed in a trial of idasanutlin and venetoclax, which yielded response rates of ~50% in relapsed/refractory AML and was, after dose adjustments, reasonably well tolerated.^29^

The concordance of these results may indicate that inhibition of MDM2 alone is insufficient to prevent downregulation of p53, as there are multiple other oncogenic pathways and mechanisms involved in its regulation.^9^ MDM4 (MDMX) is also a negative regulator of p53 that is often overexpressed in AML.^30^ It has been suggested that maintained activity of MDM4 in the presence of MDM2 inhibition could constitute a form of resistance.^31^ Preclinical studies suggest that inhibition of both MDM2 and MDM4 may stabilize p53 in leukemic cells;^32^ however, no clinical data for dual inhibition of MDM2/MDM4 in AML patients have been published.

In vitro studies have shown that MDM2 inhibition is associated with the selection, but not induction, of *TP53*‐mutated clones in patients with myeloproliferative neoplasms.^33^ A relationship between poor response to MDM2 inhibition and the emergence of mutant *TP53* has previously been demonstrated in AML.^16^, ^34^ In the present study, *TP53* mutations were detected by TP53 PCR/NGS or Archer myeloid panel sequencing during treatment in four participants (7%) receiving milademetan monotherapy, two of whom had a CR and the other two treatment failure. Samples available from two of these participants analyzed by a more sensitive ddPCR demonstrated the emergent *TP53* mutants as expanded pre‐existing mutant clones; however, none emerged in the combination treatment arm, despite long exposure to treatment in some participants, suggesting combination treatment may mitigate this common mechanism of secondary resistance. One study has shown that expansion of p53 mutant subclones was reversible in patients with myeloproliferative neoplasms when the MDM2 inhibitor was discontinued in 4/5 patients,^35^ although the fate of these cells was unclear. However, this well‐documented phenomenon suggests that not all *TP53* mutant subclones are truly leukemic but may rather represent clonal hematopoiesis of indeterminate potential mutations that do not necessarily evolve into fully transformed p53 mutant AML cells. Ongoing single‐cell analyses may illuminate this issue.

Despite the low clinical response rate to milademetan in patients with AML and high‐risk MDS, serum MIC‐1 was induced during treatment at all dose levels and increased with increasing milademetan exposure. As MIC‐1 is known to be a direct biomarker of p53 activation,^25^ this confirms the pharmacologic effect of milademetan. However, it may not represent a marker for p53‐activation induced apoptosis in tumor cells. Moreover, it is not known whether a certain amount of MIC‐1 induction, or duration of MIC‐1 induction, predicts clinical benefit.

Milademetan *C*_max_, AUC_0‐24h_, and AUC_last_ increased in a dose‐proportional manner in both monotherapy and combination arms, as has been observed previously.^26^ Notably, for Cohorts 1 through 3, in which milademetan was administered in a similar dosing schedule to the previous study, *T*_max_ was similar (~3 h), and at Cycle 1 Day 15 in the present study, the AR was 1.21, compared with 1.18 to 3.11 at Cycle 1 Day 21 in the previous study.

The key limitation of this study was the small number of participants in the milademetan‐AZA combination treatment arm, which may have precluded evidence of response, or alternatively, the potential suppression of mutant *TP53* selection and clonal expansion over time in responding participants. Further research into whether the combination of an MDM2 inhibitor and a hypomethylating agent may suppress p53 mutant clones by activating other tumor suppressors (eg, p73) is warranted; studies have suggested that downregulation of p53 in AML cells may result in a functional switch to p73.^36^ Sequencing plays a key role; hypomethylating agent activity is mostly dependent on the S phase of the cell cycle,^37^ whereas MDM2 inhibition is largely associated with G1/S arrest.^38^, ^39^ Further optimization of AZA and milademetan dose schedules could enhance response while reducing the frequency of adverse events.

In conclusion, milademetan demonstrated minimal clinical activity in heavily pre‐treated patients with AML/MDS as monotherapy and in combination with AZA. Future studies utilizing milademetan combinations with 1 or more agents with different modes of action are warranted.

## AUTHOR CONTRIBUTIONS

**Courtney D. DiNardo:** Conceptualization (equal); investigation (equal); writing – original draft (equal); writing – review and editing (equal). **Rebecca Olin:** Investigation (equal); writing – review and editing (equal). **Eunice S. Wang:** Investigation (equal); writing – review and editing (equal). **Barry Skikne:** Investigation (equal); writing – review and editing (equal). **Joseph Rosenthal:** Investigation (equal); writing – review and editing (equal). **Prasanna Kumar:** Conceptualization (equal); data curation (equal); formal analysis (equal); investigation (equal); writing – original draft (equal); writing – review and editing (equal). **Hiroyuki Sumi:** Investigation (equal); writing – review and editing (equal). **Yoshiyuki Hizukuri:** Investigation (equal); writing – review and editing (equal). **Ying Hong:** Investigation (equal); writing – review and editing (equal). **Parul Patel:** Investigation (equal); writing – review and editing (equal). **Takahiko Seki:** Investigation (equal); writing – review and editing (equal). **Tao Duan:** Formal analysis (equal); writing – review and editing (equal). **Arnaud Lesegretain:** Conceptualization (equal); writing – review and editing (equal). **Michael Andreeff:** Conceptualization (equal); data curation (equal); writing – review and editing (equal).

## FUNDING INFORMATION

This study was funded by Daiichi Sankyo Inc.

## CONFLICT OF INTEREST STATEMENT

CDD has received research support and/or consulting fees from Abbvie, Astellas, Astex, Beigene, BMS, Cleave, Daiichi Sankyo, Foghorn, Genetech, GenMab, Gilead, GSK, Immunogen, ImmuneOnc, Jazz, Leukemia and Lymphoma Society, Loxo, Novartis, Notable Labs, Rigel, Servier; RO has received support and/or consulting fees from Actinium, Cellectis, Rigel, and Servier; ESW has received advisory board and/or consulting fees from Abbvie, BMS, CTI Biopharma, Daiichi Sankyo, Genentech, GSK, Johnson & Johnson, Kite, Kura, Novartis, Rigel, Sellas, and Sumitomo Pharma; speaker fees from Astellas, Kura, and Pfizer; and participation fees from Abbvie and Gilead; BS is an investigator in the study and in so doing, payment for the cost of conduct of the study as well as receipt of the investigative agents were made to the Clinical Cancer Research Institute at the University of Kansas Cancer Center; JR has no competing interests related to this manuscript; MA has received research support, consulting and/or advisory fees from Amgen, AstraZeneca, Breast Cancer Research Foundation, Cancer Prevention and Research Institute of Texas, Celegene, Daiichi Sankyo, Jazz Pharmaceuticals, Karyopharm Therapeutics, National Institutes of Health/National Cancer Institute, Ono Pharmaceutical, Reata Pharmaceuticals, Senti Biosciences, United Therapeutics; travel expenses from Amgen, Aptose Biosciences, AstraZeneca, Oncoceutics; holds a patent with Reata Pharmaceuticals; and holds stock and other ownership interests in Aptose Biosciences, Eutropics, Oncoceutics, Oncolyze; PK, Y Hong, PP, TS, TD and AL are employed at Daiichi Sankyo Inc, the study sponsor, and HS and Y Hizukuri are employed at Daiichi Sankyo Co, Ltd, Japan, and all own company stocks.

## ETHICS STATEMENT

This study was conducted in accordance with Good Clinical Practice guidelines and all applicable regulatory requirements, including the principles of the Declaration of Helsinki and International Conference on Harmonization guidelines. The study protocol and informed consent procedures were approved the United States Food and Drug Administration and local institutional review boards.

## CONSENT

Study subjects freely gave written consent after adequate explanation of the aims, methods, anticipated benefits, and potential hazards of the study and before any protocol‐specific procedures or administration of study drugs. Subjects were given the opportunity to ask questions and receive responses with adequate time to deliberate prior to study participation.

## CLINICAL TRIAL REGISTRATION

NCT02319369.

## Supporting information


Figure S1.

Figure S2.

Figure S3.

Figure S4.

Figure S5.

Table S1.

## Data Availability

Anonymized individual participant data (IPD) on completed studies and applicable supporting clinical study documents may be available upon request at https://vivli.org/. In cases where clinical study data and supporting documents are provided pursuant to our company policies and procedures, Daiichi Sankyo Companies will continue to protect the privacy of company and our clinical study subjects. Details on data sharing criteria and the procedure for requesting access can be found at this web address: https://vivli.org/ourmember/daiichi‐sankyo/.
