# Supplementary material for: Phase 1 dose escalation study of the MDM2 inhibitor milademetan as monotherapy and in combination with azacitidine in patients with myeloid malignancies
Source: Cancer Med. 2024 Jul 19;13(14):e70028. doi: 10.1002/cam4.70028 (PMC11258486; doi:10.1002/cam4.70028)
Supplement: Supplementary file 1 — Figure S1. [file CAM4-13-e70028-s002.pdf]

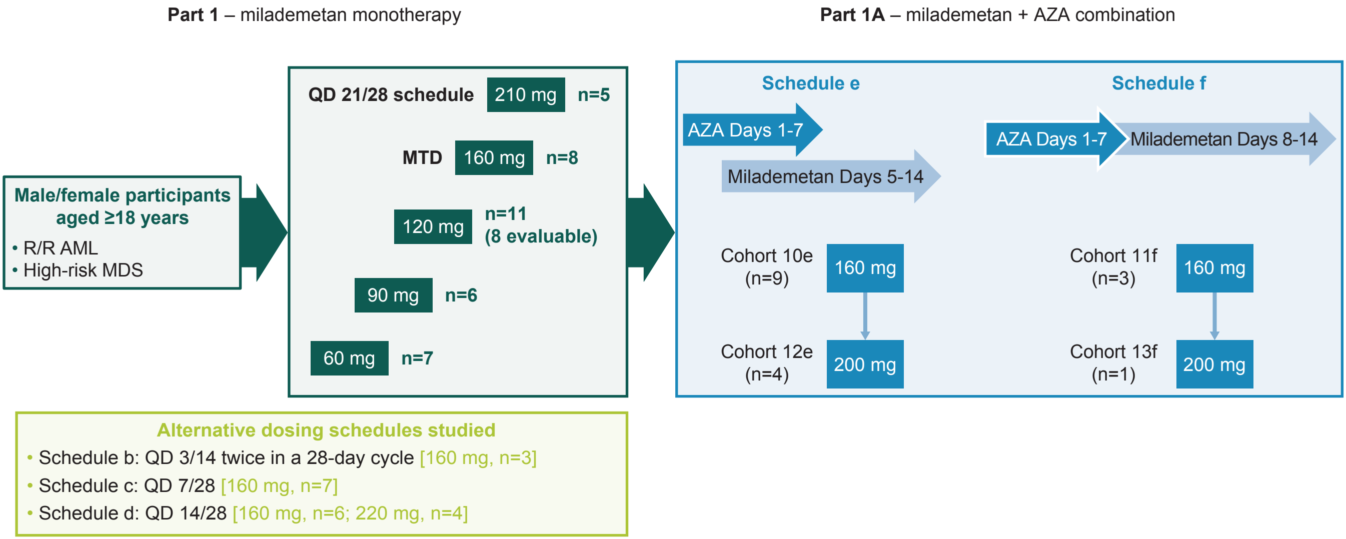

Part 1 – milademetan monotherapy
Part 1A – milademetan + AZA combination
Male/female participants aged ≥18 years
• R/R AML
• High-risk MDS
QD 21/28 schedule
210 mg
n=5
MTD
160 mg
n=8
120 mg
n=11 (8 evaluable)
90 mg
n=6
60 mg
n=7
Schedule e
AZA Days 1-7
Milademetan Days 5-14
Cohort 10e (n=9)
160 mg
Cohort 12e (n=4)
200 mg
Schedule f
AZA Days 1-7
Milademetan Days 8-14
Cohort 11f (n=3)
160 mg
Cohort 13f (n=1)
200 mg
Alternative dosing schedules studied
• Schedule b: QD 3/14 twice in a 28-day cycle [160 mg, n=3]
• Schedule c: QD 7/28 [160 mg, n=7]
• Schedule d: QD 14/28 [160 mg, n=6; 220 mg, n=4]
