# Supplementary material for: Phase 1 dose escalation study of the MDM2 inhibitor milademetan as monotherapy and in combination with azacitidine in patients with myeloid malignancies
Source: Cancer Med. 2024 Jul 19;13(14):e70028. doi: 10.1002/cam4.70028 (PMC11258486; doi:10.1002/cam4.70028)
Supplement: Supplementary file 2 — Figure S2. [file CAM4-13-e70028-s001.pdf]

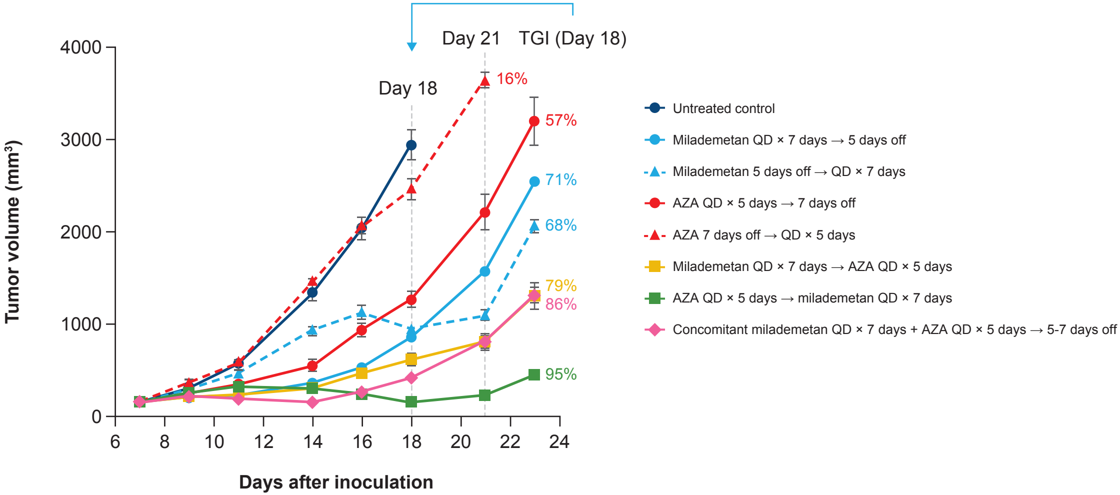

TGI (Day 18)
Day 21
Day 18
16%
57%
71%
68%
79%
86%
95%
4000
3000
2000
1000
0
6
8
10
12
14
16
18
20
22
24
Tumor volume (mm³)
Days after inoculation
Untreated control
Milademetan QD × 7 days → 5 days off
Milademetan 5 days off → QD × 7 days
AZA QD × 5 days → 7 days off
AZA 7 days off → QD × 5 days
Milademetan QD × 7 days → AZA QD × 5 days
AZA QD × 5 days → milademetan QD × 7 days
Concomitant milademetan QD × 7 days + AZA QD × 5 days → 5-7 days off
