# Supplementary figures and images for: Phase 1 dose escalation study of the MDM2 inhibitor milademetan as monotherapy and in combination with azacitidine in patients with myeloid malignancies
Source: Cancer Med. 2024 Jul 19;13(14):e70028. doi: 10.1002/cam4.70028 (PMC11258486; doi:10.1002/cam4.70028)

**a**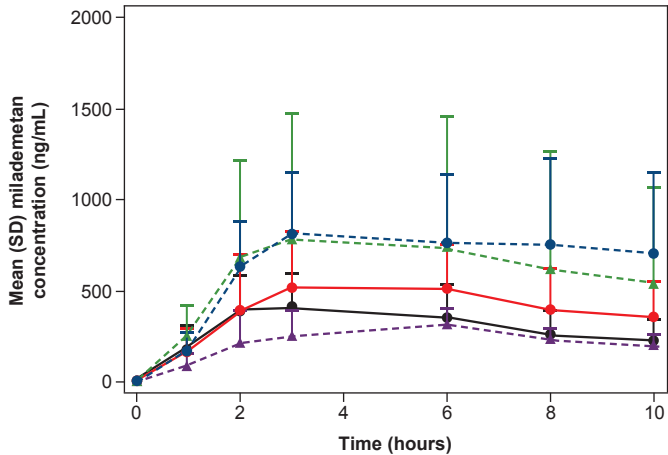**b**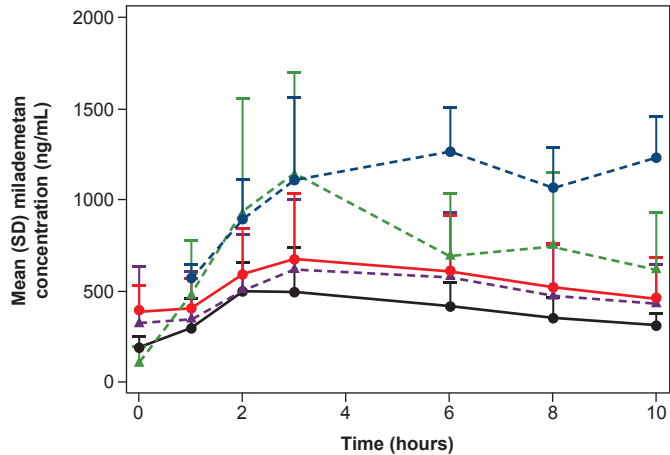

● Cohort 1 (60 mg QD 21/28)    ▲ Cohort 2 (90 mg QD 21/28)    ● Cohort 3 (120 mg QD 21/28)    ▲ Cohort 4 (160 mg QD 21/28)    ● Cohort 5 (210 mg QD 21/28)

Supplement: Supplementary file 3 — Figure S3. [file CAM4-13-e70028-s004.pdf]

**a**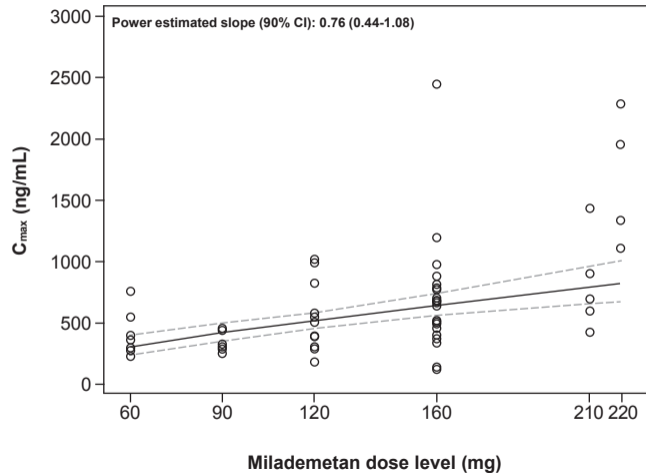**b**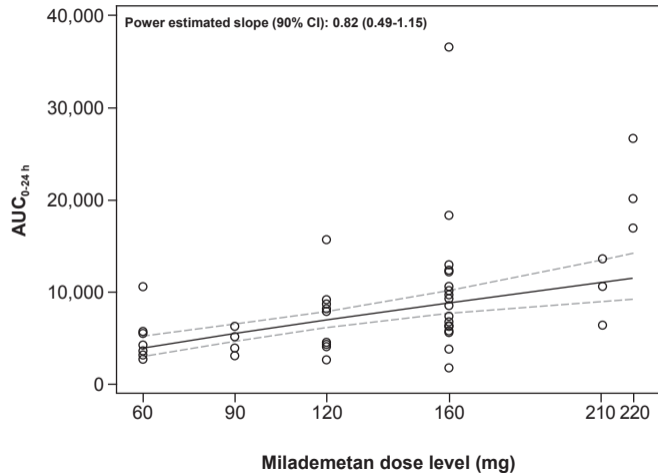

Supplement: Supplementary file 4 — Figure S4. [file CAM4-13-e70028-s005.pdf]
