# Supplementary material for: Phase 1 dose escalation study of the MDM2 inhibitor milademetan as monotherapy and in combination with azacitidine in patients with myeloid malignancies
Source: Cancer Med. 2024 Jul 19;13(14):e70028. doi: 10.1002/cam4.70028 (PMC11258486; doi:10.1002/cam4.70028)
Supplement: Supplementary file 5 — Figure S5. [file CAM4-13-e70028-s003.pdf]

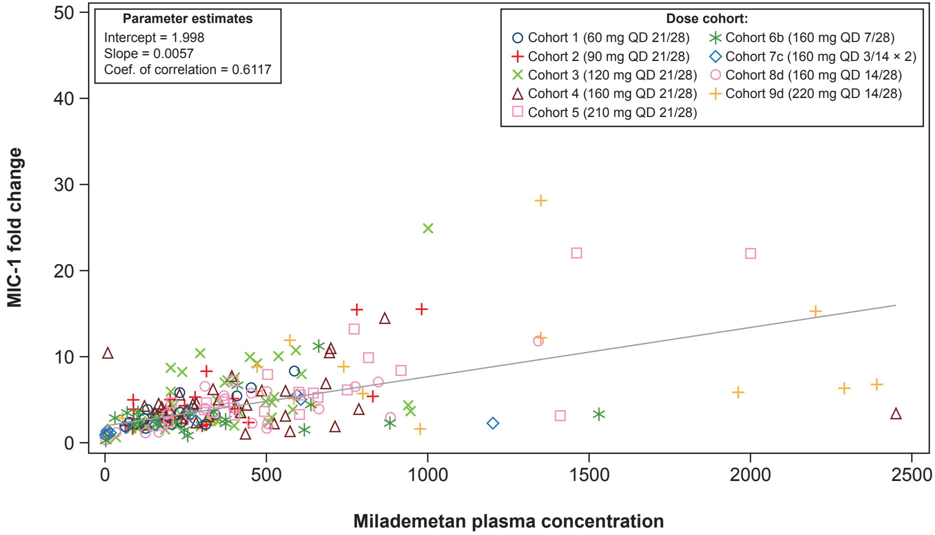

Parameter estimates
Intercept = 1.998
Slope = 0.0057
Coef. of correlation = 0.6117
Dose cohort:
Cohort 1 (60 mg QD 21/28)
Cohort 2 (90 mg QD 21/28)
Cohort 3 (120 mg QD 21/28)
Cohort 4 (160 mg QD 21/28)
Cohort 5 (210 mg QD 21/28)
Cohort 6b (160 mg QD 7/28)
Cohort 7c (160 mg QD 3/14 × 2)
Cohort 8d (160 mg QD 14/28)
Cohort 9d (220 mg QD 14/28)
MIC-1 fold change
Miladematan plasma concentration
0
10
20
30
40
50
0
500
1000
1500
2000
2500
