# Supplementary material for: Phase 1 dose escalation study of the MDM2 inhibitor milademetan as monotherapy and in combination with azacitidine in patients with myeloid malignancies
Source: Cancer Med. 2024 Jul 19;13(14):e70028. doi: 10.1002/cam4.70028 (PMC11258486; doi:10.1002/cam4.70028)
Supplement: Supplementary file 6 — Table S1. [file CAM4-13-e70028-s006.docx]

# Supplementary Material

# Supplementary Table 1. Dose-limiting Toxicities (DLT Evaluable Set)^a^

|  | **Milademetan  monotherapy** | | | | **Milademetan + AZA** |
| --- | --- | --- | --- | --- | --- |
|  | **Cohort 1 (N=5)** | **Cohort 4 (N=8)** | **Cohort 5 (N=5)** | **Cohort 9d (N=3)** | **Cohort 12e (N=4)** |
| Milademetan dosing regimen | 60 mg QD 21/28 | 160 mg QD 21/28 | 210 mg QD 21/28 | 220 mg QD 14/28 | 200 mg QD 10/28 |
| Participants with TEAE classified as DLT, n (%) | 1 (20.0) | 2 (25.0) | 3 (60.0) | 2 (66.7) | 2 (50.0) |
| Nausea | 0 | 0 | 1 (20.0) | 2 (66.7) | 0 |
| Fatigue | 0 | 0 | 1 (20.0) | 0 | 2 (12.5) |
| Cellulitis | 0 | 0 | 1 (20.0) | 0 | 0 |
| Diarrhea | 0 | 1 (12.5) | 0 | 0 | 0 |
| Hypokalemia | 0 | 1 (12.5) | 0 | 0 | 0 |
| Renal failure | 0 | 0 | 1 (20.0) | 0 | 0 |
| Vomiting | 1 (20.0) | 0 | 0 | 0 | 0 |
| Syncope | 0 | 0 | 0 | 0 | 1 (6.3) |

DLT, dose-limiting toxicity; QD, once daily; TEAE, treatment-emergent adverse events.

^a^DLT were not reported in Cohorts 2, 3, 6, 7, 8, 10, 11 or 13.

# Supplementary Table 2. Summary of Treatment-emergent Adverse Events (Safety Analysis Set)

| **TEAE, n (%)** | **Milademetan monotherapy (N=57)** | **Milademetan  + AZA  (N=17)** |
| --- | --- | --- |
| Any TEAE | 57 (100.0) | 17 (100.0) |
| Grade ≥3 TEAE | 48 (84.2) | 16 (94.1) |
| SAE | 39 (68.4) | 16 (94.1) |
| TEAE associated with milademetan discontinuation | 13 (22.8) | 4 (23.5) |
| TEAE associated with AZA discontinuation | - | 3 (17.6) |
| TEAE associated with death | 9 (15.8)^a^ | 5 (29.4)^b^ |
| Milademetan-related TEAE | 47 (82.5) | 11 (64.7) |
| Grade ≥3 TEAE | 19 (33.3) | 5 (29.4) |
| SAE | 7 (12.3) | 4 (23.5) |
| TEAE associated with milademetan discontinuation | 5 (8.8) | 3 (17.6) |
| TEAE associated with AZA discontinuation | - | 2 (11.8) |
| TEAE associated with death | 0 | 0 |
| AZA-related TEAE | - | 15 (88.2) |
| Grade ≥3 | - | 7 (41.2) |
| SAE | - | 3 (17.6) |
| TEAE associated with milademetan discontinuation | - | 3 (17.6) |
| TEAE associated with AZA discontinuation | - | 2 (11.8) |
| TEAE associated with death | - | 0 |

AZA, azacytidine; SAE, serious adverse event; TEAE, treatment-emergent adverse events.

^a^Events associated with a fatal outcome included sepsis (4 patients), lung infection (2 patients); intracranial hemorrhage, multi-organ failure, pneumonia, and respiratory failure each reported 1 patient. None of these events or deaths were assessed by the investigator as related to milademetan.

^b^Events associated with fatal outcome included death (2 patients); cardiac failure, failure to thrive, and bacterial pneumonia each reported in 1 patient. None of these deaths were assessed by the investigator as related to milademetan or AZA.

**Supplementary Table 3. Baseline Characteristics and *TP53* Mutation Status of Participants With Treatment Response or Stable Disease**

| **Participant ID** | **Treatment group** | **Treatment response** | **Sex/age** | **Diagnosis** | **Cytogenetics** | **Risk stratification** | **Prior therapy** | **Baseline *TP53* mutations** | **Emergent *TP53* mutations** |
| --- | --- | --- | --- | --- | --- | --- | --- | --- | --- |
| 1001-1005 | Milademetan monotherapy | CR | F/71 | AML | 46,XX[30].nuc ish(CEP8x3)[2/400],(RUNX1T1x3)[1/400] | Adverse | CLO, CYT | No | Yes |
| 1002-1013 | Milademetan monotherapy | CR | M/79 | AML | 46, XY, t (X; 18; q11.2; q12) [1]/46, XY [19] | Intermediate | DEC | No | Yes |
| 1002-1017 | Milademetan monotherapy | Cri | F/80 | AML | 46, XX, +8 [4]/46, XX [16] | Intermediate | RIG, AZA, DEC | Yes | No |
| 1001-1007 | Milademetan monotherapy | mCR | M/78 | MDS | 46,XY[20] | >4.5-6: High | AZA | No | No |
| 1001-1017 | Milademetan monotherapy | SD | M/75 | MDS | 46,XY[28].nuc ish(D5S721,EGR1)x2[195],(D7 Z1,D7S486)x2[198],  (CEP8x2)[193],(D20S108x2) [197] | >4.5-6: High | AZA, LEN, DEC | No | No |
| 1002-1048 | Milademetan + AZA | Cri | F/76 | AML | 46,XX,del(7)(q22q34)[15]/46,idem,t(5 ;12)(q31 ;p13)[2]/46,XX[3] | Adverse | CYT, HU, HCB, VEN, IDA, CLA, MID, DEC | No | No |
| 1002-1049 | Milademetan + AZA | Cri | M/82 | AML | 46,XY,del(5)(q13q33)[3]/46,idem,+1,der(1 ;21)(q10 ;q10)[4]/46,XY,+1,der(1 ;21)(q10 ;q10)[10]/46,XY[3] | Adverse | AZA, VEN | No | No |
| 1004-1005 | Milademetan + AZA | MLFS | M/70 | AML | 46,XY,del9(q13-q22)[2]/46,XY[18] | Intermediate | CYT, DAU, FLU, DEX, DEC, VEN | No | No |
| 1004-1006 | Milademetan + AZA | MLFS | F/64 | AML | 46,XX,del(7)(q22)[16]/46XX[4] | Adverse | DAU, CYT, VEN, DEC | No | No |

AZA, azacytidine; CLA, cladribine; CLO, clofarabine; CR, complete remission; Cri, complete remission with incomplete hematological recovery; CYT, cytarabine; DAU, daunorubicin; DEC, decitabine; DEX, dexamethasone; FLU, fludarabine; HCB, hydrocarbazine; HU, hydroxyurea; IDA, idarubicin; LEN, lenalidomide; mCR, marrow complete response; MID, midostaurin; MLFS, morphologic leukemia-free state; RIG, rigosertib; SD, stable disease; VEN, venetoclax.

**Supplementary Table 4. Pre-treatment and Post-treatment *TP53* Mutations**

| **Treatment** | **No. pts at baseline** | **No. pts post-baseline** | **Baseline *TP53* mutations** | **Post-baseline *TP53* mutations** | | |
| --- | --- | --- | --- | --- | --- | --- |
|  |  |  |  | **All** | **Persistent** | **Emergent** |
| ***TP53* genotyping by PCR/NGS** | | | | | | |
| Milademetan monotherapy | 56 | 18 | 1 | 4 | 1 | 3 |
| **Archer Myeloid panel** | | | | | | |
| Milademetan monotherapy | 47 | 21 | 1 | 5 | 1 | 4^a^ |
| Milademetan + AZA | 17 | 16 | 1 | 1 | 1 | 0 |
| **ddPCR assay** | | | | | | |
| Milademetan monotherapy |  |  | 2 | 2 | 2 | 0 |

AZA, azacytidine; ddPCR, digital droplet polymerase chain reaction; NGS, next generation sequencing; PCR, polymerase chain reaction; pts, patients.

^a^Archer panel sequencing identified a TP53 mutation in 1 more participant than that identified by the previous PCR/NGS.

**Supplementary Figure 1. Study Design**


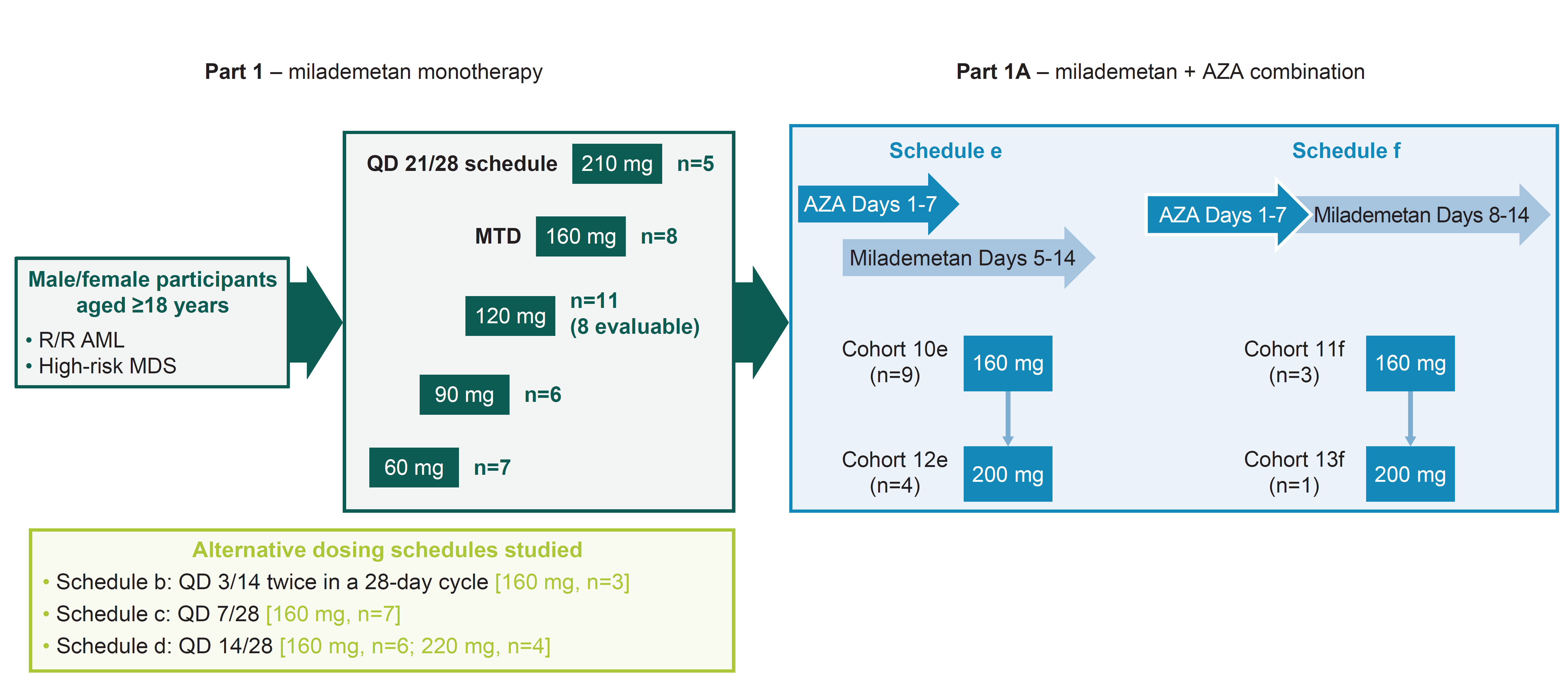


AZA, azacytidine; MDS, myelodysplastic syndrome; QD, once daily; R/R AML, relapsed/refractory acute myeloid leukemia.

**Supplementary Figure 2. Tumor Growth Inhibition by Concurrent Versus Sequential Treatment of AZA and Milademetan**


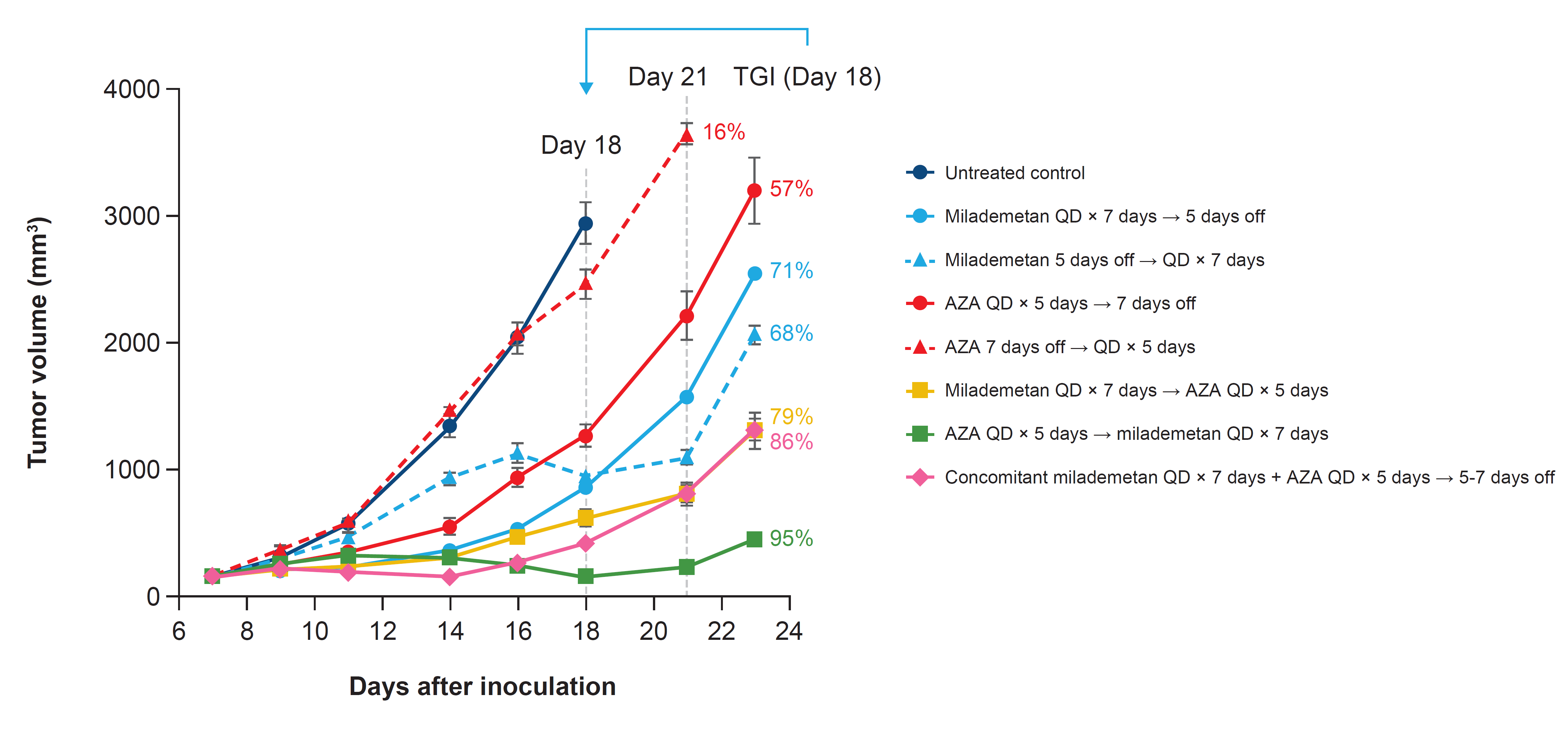


AZA, azacytidine; QD, once daily.

Milademetan was orally administered once daily for 7 days at 50 mg/kg as a salt-free form, and azacitidine was intravenously administered once daily for days at 4 mg/kg to mice bearing the xenografts of MOLM-13 human AML cell line. Each data point and bar represents the mean and standard error of the estimated tumor volume in each group, respectively (N=6).

# Supplementary Figure 3. Mean Milademetan Plasma Concentration Over Time on (A) Day 1 and (B) Day 15 With Milademetan Dosing for 21 Days of the First 28-day Cycle (Linear Scale)

| 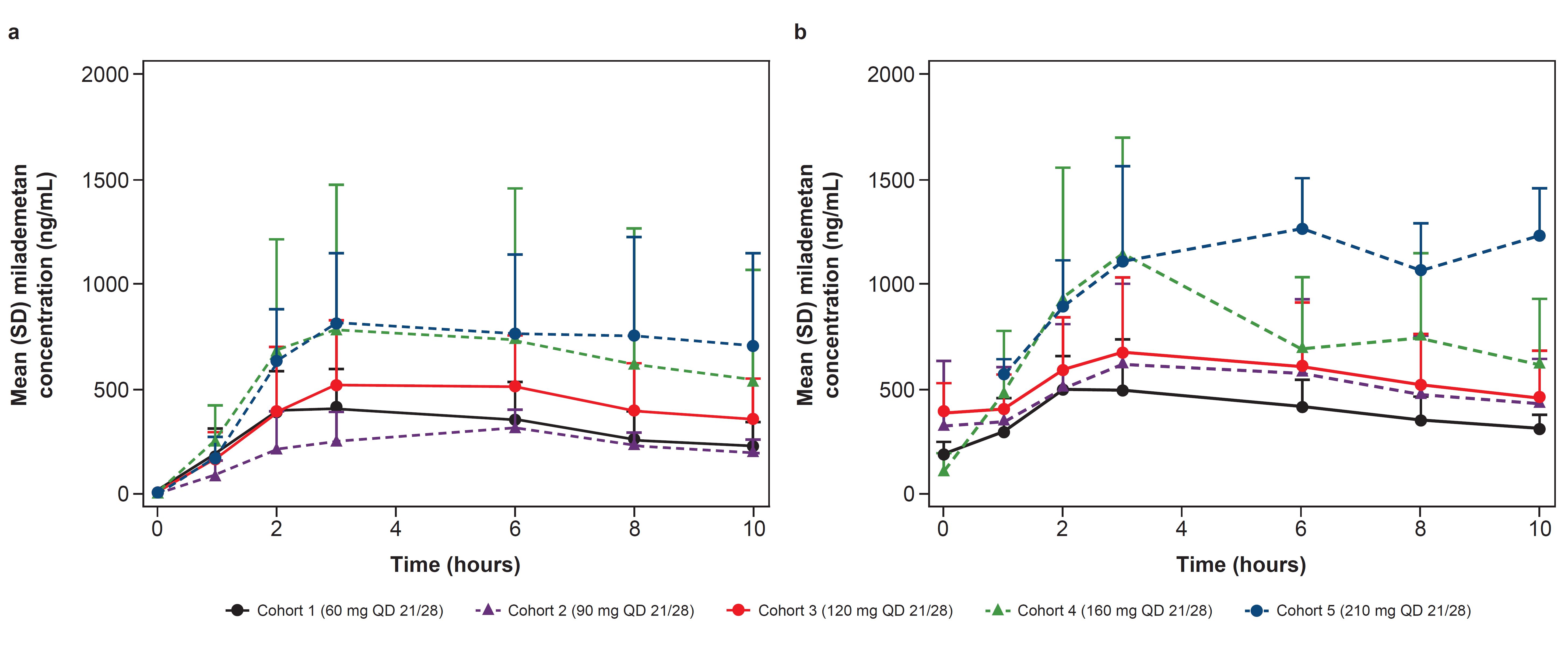 |  |
| --- | --- |

QD, once daily; SD, standard deviation.

# Supplementary Figure 4. Milademetan C_max_ (A) and AUC_0-24h_ (B) on Day 1 of Cycle 1 With Milademetan Monotherapy


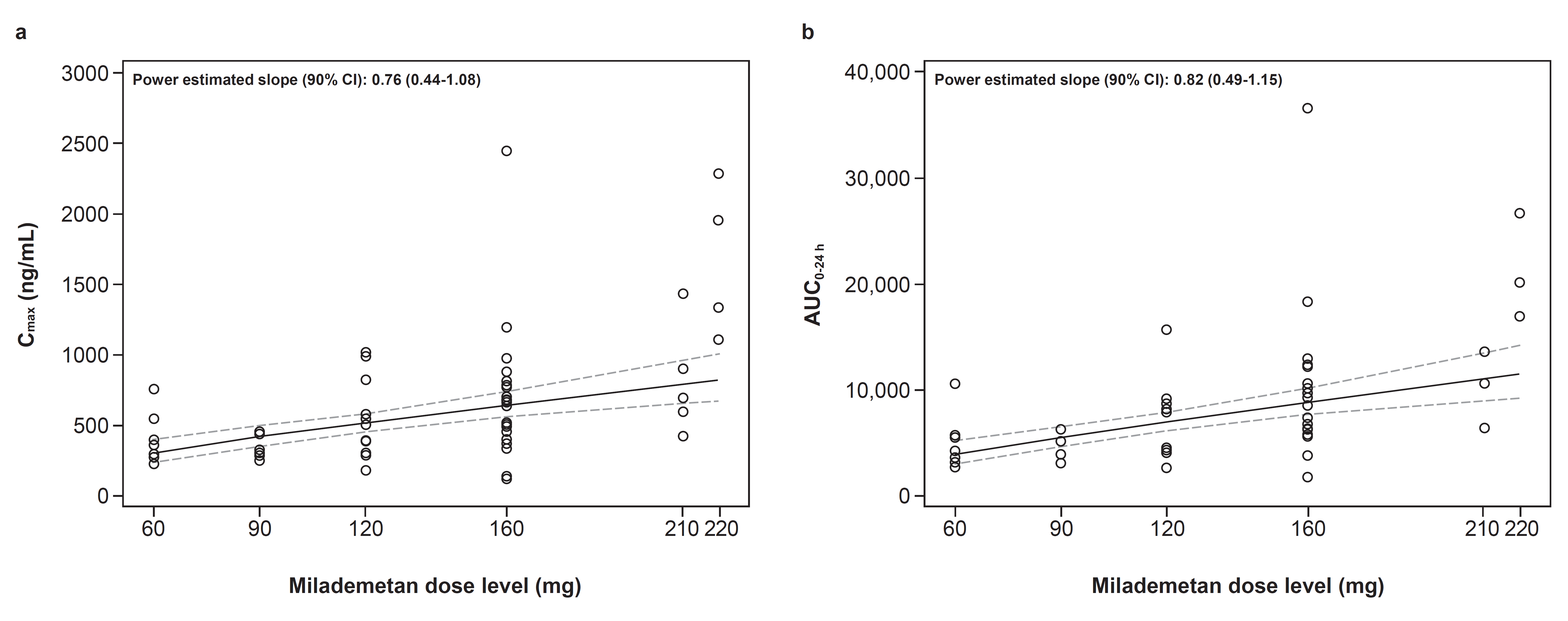


AUC_0-24h_, area under the plasma concentration-time curve up to 24 hours; CI, confidence interval; C_max_, maximum plasma concentration.

# Supplementary Figure 5. MIC-1 Fold Change and Time-matched Milademetan Plasma Concentration by Dose at All Time Points With Milademetan Monotherapy

Coef, coefficient; MIC-1, macrophage inhibitory cytokine 1; QD, once daily.
